# A cross-sectional study of knife injuries at a London major trauma centre

**DOI:** 10.1308/003588414X13824511649616

**Published:** 2014-01

**Authors:** JR Pallett, E Sutherland, E Glucksman, M Tunnicliff, JW Keep

**Affiliations:** King’s College Hospital NHS Foundation Trust,UK

**Keywords:** Epidemiology, Violence, Trauma, Non-accidental

## Abstract

**INTRODUCTION:**

No national recording systems for knife injuries exist in the UK. Understanding the true size and nature of the problem of knife injuries is the first stage in reducing the burden of this injury. The aim of this study was to survey every knife injury seen in a single inner city emergency department (ED) over a one-year period.

**METHODS:**

A cross-sectional observational study was performed of all patients attending with a knife injury to the ED of a London major trauma centre in 2011. Demographic characteristics, patterns of injury, morbidity and mortality data were collected.

**RESULTS:**

A total of 938 knife injuries were identified from 127,191 attendances (0.77% of all visits) with a case fatality rate of 0.53%. A quarter (24%) of the major trauma team’s caseload was for knife injuries. Overall, 44% of injuries were selfreported as assaults, 49% as accidents and 8% as deliberate self-harm. The highest age specific incident rate occurred in the 16–24 year age category (263/100,000). Multiple injuries were seen in 19% of cases, of which only 81% were recorded as assaults. The mean length of stay for those admitted to hospital was 3.04 days. Intrathoracic injury was seen in 26% of cases of chest trauma and 24% of abdominal injuries had a second additional chest injury.

**CONCLUSIONS:**

Violent intentional injuries are a significant contributory factor to the workload of the major trauma team at this centre. This paper contributes to a more comprehensive understanding of the nature of these injuries seen in the ED.

Knife injuries have become a significant public concern in the UK with apparent rising incidence observed by emergency trauma services in London.^1,2^ Between 1997 and 2005, the number of people admitted to hospital with a ‘sharp object’ increased by 30%.^3^ In England and Wales during 2011–2012, 200 homicides, 246 attempted murders and 4,490 admissions to hospital due to a sharp object were officially recorded.^4^

The annual cost to the National Health Service for all violent injury against adults has been estimated at £2.2 billion per year.^5^ Based on the Trauma Audit and Research Network (TARN) database, the costs of penetrating trauma per patient in England and Wales have been estimated at approximately £8,000.^6^ Published by the Department of Health in 2012, *Protecting People, Promoting Health* advocates a public health approach to violence prevention for England.^7^ The Health Development Agency recommended that a health needs assessment begins with establishing the size and nature of the problem.^8^ For knife injuries, a comprehensive understanding of who gets injured when and how is therefore required in order to inform future evidence-based violence prevention strategies.

No national routine surveillance system exists for all knife injuries. The TARN has data entry criteria for the most serious cases including hospital admission for longer than 72 hours. Hospital Episode Statistics (HES) also records national data but is limited by formal admission to hospital data only. Detailed descriptive studies from ad hoc surveys based in UK EDs have previously been published but are limited by non-standardised case definitions of ‘penetrating trauma’ and ‘sharp objects’.^9–13^

Common perceptions among ED practitioners are that these injuries are substantially more prevalent than reported within existing systems, and that true ‘assaults’ are often concealed and described as ‘accidents’ to avoid police involvement. In addition, selective high profile media and political attention to mortality cases alone risks not accurately understanding true prevalence trends. These differences have not been quantified previously with ED data.

We hypothesised that a more meaningful analysis of the burden and trends of knife injuries requires a detailed understanding of all cases that present to the ED. The aim of this study was therefore to analyse all such injuries that present to a London major trauma centre (MTC) ED. The objectives were to describe the epidemiological characteristics, injury patterns, morbidity, mortality and disposition of all knife injuries that presented during 2011.

## Methods

A single centre cross-sectional observational study is described of all knife injuries presenting to an ED in 2011. The study was carried out at King’s College Hospital, the MTC for the South East London trauma network, between 1 January and 31 December 2011. Cases were defined with the standard dictionary definition of a knife as ‘an instrument composed of a blade fixed into a handle, used for cutting or as a weapon’. Other sharp objects causing penetrating injury such as scissors, glass, bottles, industrial tools and gunshot wounds were excluded. Reattendances for complications or follow-up of the original injury were also excluded. Cases were identified through the local electronic health record system and notes reviewed for eligibility to the study.

Data were collected on demographics, anatomical region of injury, investigations, injury sustained and length of hospital stay (LOS). Statistical analyses were carried out using the chi-squared test for association between categorical variables and the z-test for differences in proportions. Statistical significance was determined at the 5% level. Approval was obtained from the local clinical effectiveness unit and the clinical governance lead prior to commencing the study. Further ethical committee approval was not required.

## Results

### Epidemiology of knife injuries

**Figure 1:**
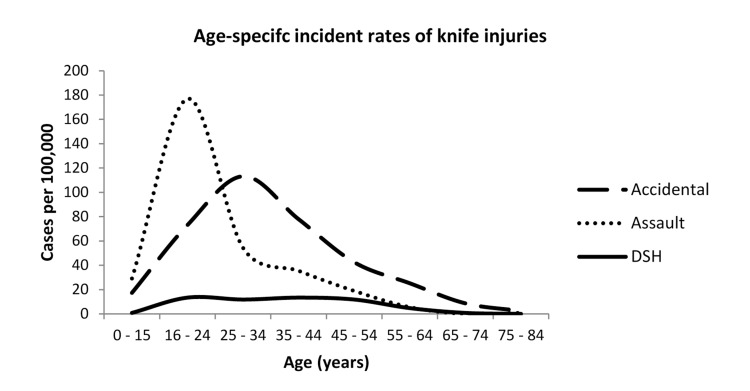
Age specific incident rates of knife injuries

**Figure 2:**
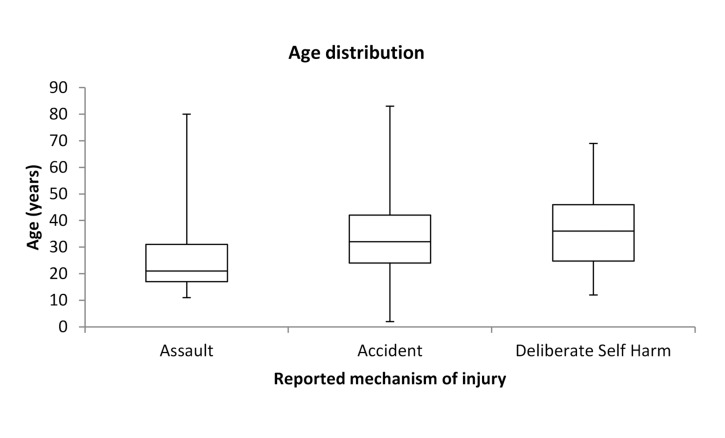
Age distribution by reported mechanism of injury

A total of 127,191 attendances were recorded in the 12-month study period with 938 cases of knife injury seen in the ED (0.77% of all ED visits). Table 1 shows the baseline characteristics by mechanism of injury reported to clinician. A quarter (24%) of all cases activating the MTC were attributed to knife injuries and knives accounted for 94.1% of cases of major penetrating trauma. The crude annual incidence rate seen in the ED population was 737/100,000 persons with age specific rates highest (263/100,000) in the 16–24 years category (Fig 1). A similar demographic profile for age range and sex was seen in injuries reported as ‘accidents’ (Fig 2).

**Table 1:** Baseline characteristics by mechanism of injury reported to clinician

	Assaults	Accidents	Deliberate self-harm
**Total**	**408 (43%)**	**458 (49%)**	**72 (8%)**
Median age (range) in years	21 (12–80)	32 (2–83)	36 (12–69)
Male	375 (91%)	313 (68%)	34 (47%)
Presentation: 8am – 4.59pm	93 (23%)	245 (54%)	24 (33%)
Monthly variation	No	Yes (*p*<0.05)	No
Diurnal variation	Yes (p<0.001)	No	No

Accidents were associated with month of the year (*p*<0.05) with the highest number seen in July and the lowest in February. There was no such association observed for reported assaults or self-harm with the month of the year. Assaults were overall most likely to be seen on a Saturday (*p*<0.05). A linear correlation was observed for all injuries being associated with longer days and higher temperatures but this trend did not reach statistical significance.

### Patterns of injury and morbidity

A fifth (18.9%) of all cases were multiple injuries and 81.4% of these were reported as assaults.

*Abdominal trauma:* One hundred and forty cases were identified and 65.0% were isolated injuries. A quarter (24.4%) of all abdominal injuries had an associated chest injury. The most commonly injured intra-abdominal structures were the small and large intestine (35.4%) liver (25.0%) and spleen (8.3%) (Table 2).

**Table 2:** Injuries associated with abdominal trauma identified by computed tomography or at laparotomy

Abdominal injuries (*n*=140)	
Superficial	82 (58.6%)
Hollow viscus (stomach/small/large intestine)	19 (13.6%)
Solid organ (spleen/liver/kidney)	15 (10.7%)
Peritoneum breached but no specific injury identified	12 (8.6%)
Abdominal wall haematoma	10 (7.1%)
Source of haemorrhage unidentified	2 (1.4%)

*Chest trauma:* One hundred and ninety chest injuries were identified, 60.0% of which were isolated. Almost a fifth (17.9%) of chest injuries were associated with a second abdominal injury. Over a quarter (26.3%) penetrated the pleura into the thoracic cavity.

*Limb trauma:* A total of 662 limb injuries were identified. The vast majority (91.1%) of these were isolated; 90.9% were superficial injuries without evidence of neurovascular injury and required dressing or closure with local anaesthetic in the ED, 2.4% had examination under general anaesthesia and a further 5.6% required evaluation by the plastic surgery team for suspected tendon injury.

**Figure 3:**
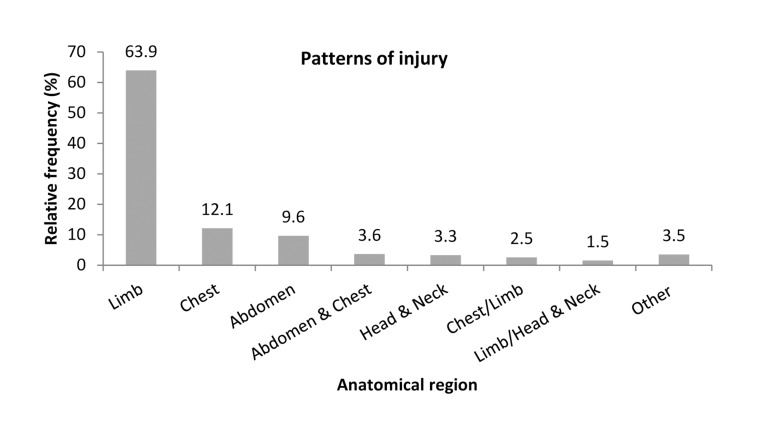
Anatomical patterns of injury

*Head and neck trauma:* Sixty-two cases were identified of which 50.0% were isolated injuries. Over a fifth (22.6%) required examination under anaesthesia (Fig 3).

### Disposition and length of stay

Excluding transfers to other hospitals and self-discharge, 25.7% patients were formally admitted to the hospital. The majority (70.5%) were discharged home directly from the ED. The mean LOS for those admitted was 3.04 days (range: 1–48 days). The mean LOS following a laparotomy was 9.0 days. The mean LOS for abdominal injuries with radiological signs of breach of the peritoneum and that were managed non-operatively was 5.2 days (Table 3).

**Table 3:** Disposition of cases from the emergency department

Discharged from emergency department	661 (70.5%)
Admitted directly to ward	195 (20.8%)
Referred for specialty review (ear, nose and throat/plastics/ophthalmology)	32 (3.4%)
Direct to theatre	27 (2.9%)
Self-discharge from emergency department	12 (1.3%)
Direct to intensive care unit	8 (0.9%)
Died in emergency department	3 (0.3%)
**Total**	**938 (100%)**

### Mortality

Four patients died, giving a case fatality rate of 0.53%. All deaths were due to penetrating cardiac injuries. Two of these patients arrived in cardiac arrest with a thoracotomy performed by the helicopter emergency medical service prior to arrival in the ED. Twenty-seven required immediate emergency surgical intervention and went directly to theatre. Fourteen had a laparotomy, four had a thoracotomy and five had both of these procedures. All of those going directly to theatre from the ED survived to discharge.

## Discussion

This is the largest UK single centre study published that exclusively describes all knife injuries seen in an ED over a single year. By analysing all injuries attributed to knives, a more detailed understanding of the true size and nature of the problem of knife injuries is presented.

Almost 1 in 5 patients with multiple injuries did not report an assault and approximately half of self-reported accidental injuries occurred to those under the age of 32 years. A similar proportion of these groups were both male and occurred out of hours. These results therefore suggest that a significant proportion of injuries classified in this survey as accidents are likely to be actual assaults although they were not reported as such at the time of injury.

Age specific injury rates in this centre are similar to those of previously published smaller studies of penetrating trauma with a median assault age of 21 years. Admission rates, however are significantly higher than comparable studies.^14,15^ This may reflect differences in patient management, the proportion of young people admitted for safeguarding reasons or particularly violent and more serious injuries at this institution. The frequent association of combined chest and abdominal injuries demonstrated in this study suggests that the injuries in this group are particularly violent, and additional injuries should be anticipated on arrival in the ED. The observed mortality rate in this cohort was low. This may reflect a low case fatality ratio, expedient pathways to emergency intervention from the ED at a MTC or a selection bias in cases brought to the ED by the prehospital emergency medical services.

The strengths of this study include the comprehensive dataset by inclusion of all knife injuries over a single year. Limitations include analysis of incident rates in the ED rather than prevalence in the general population and an inner city setting of relative socioeconomic deprivation that will limit external validity. A better understanding of the patterns and burden of these injuries for the health system and society is obtained from this type of survey rather than analysis of admission and mortality data alone.

## Conclusions

Violent intentional injuries are a significant contributory factor to the workload of the MTC at this centre. This paper contributes to a more comprehensive understanding of the nature of these injuries seen in the ED.
